# Cholinergic Transmission Dysregulation and Neurodegeneration Induced by Thyroid Signaling Disruption Following Butylparaben Single and Repeated Treatment

**DOI:** 10.3390/biology14101380

**Published:** 2025-10-09

**Authors:** Paula Moyano, Andrea Flores, Javier Sanjuan, Jose Carlos Plaza, Lucía Guerra-Menéndez, Luisa Abascal, Olga Mateo, Javier del Pino

**Affiliations:** 1 Department of Pharmacology and Toxicology, Veterinary School, Complutense University of Madrid, 28040 Madrid, Spain; 2 Department of Legal Medicine, Psychiatry and Pathology, Medicine School, Complutense University of Madrid, 28041 Madrid, Spain; 3 Departement of Basic Medical Sciences, Medicine School, Universidad San Pablo-CEU, CEU Universities, Urbanización Montepríncipe, 28660 Boadilla del Monte, Spain; lguerra@ceu.es; 4 Department of Surgery, Medicine School, Complutense University of Madrid, 28040 Madrid, Spain

**Keywords:** butylparaben, SN56 basal forebrain cholinergic neurons, thyroid hormones, AChE, HSP70, NRF2 partway, Aβ, Tau

## Abstract

**Simple Summary:**

Environmental pollutants have been implicated as potential contributors to the development of Alzheimer’s disease (AD) and other neurodegenerative disorders. Among these is the widely used preservative butylparaben, which has been shown to cause neurodegeneration and cognitive deficits similar to those observed in AD. However, the precise mechanisms by which butylparaben and other environmental pollutants exert these effects remain unknown. In AD and related dementias, a selective loss of cholinergic neurons occurs in the basal forebrain. This loss leads to the denervation of key projection areas like the hippocampus and cortex, resulting in neurodegeneration in these regions and, ultimately, cognitive impairment. Our results identify alterations in specific mechanisms that may mediate butylparaben’s disruption of cholinergic transmission and its role in inducing the degeneration of basal forebrain cholinergic neurons. These findings could help explain the mechanisms behind the cognitive alterations induced by butylparaben and other pollutants that operate similarly. Ultimately, this understanding may pave the way for developing preventive and therapeutic strategies to mitigate these effects in the population.

**Abstract:**

Butylparaben (BP), a widely used preservative, was implicated in cognitive impairment, though its neurotoxic mechanisms remain elusive. Basal forebrain cholinergic neurons (BFCN) are selectively lost in dementias, contributing to cognitive decline. To explore different mechanisms related with BFCN loss, we employed BF SN56 cholinergic wild-type or silenced cells for Tau, amyloid-beta precursor protein (βApp), acetylcholinesterase (AChE), or glycogen synthase kinase-3 beta (GSK3β) genes, exposing them to BP (0.1–80 µM) for 1 or 14 days alongside triiodothyronine (T3; 15 nM), N-acetylcysteine (NAC; 1 mM), or recombinant heat shock protein 70 (rHSP70; 30 µM). BP disrupted cholinergic transmission by AChE inhibition and provoked cell death through thyroid hormones (THs) pathway disruption, Aβ/p-Tau protein accumulation, AChE-S overexpression, and oxidative stress (OS). Aβ/p-Tau accumulation was correlated with HSP70 downregulation, OS exacerbation, and GSK3β hyperactivation (for p-Tau). BP-induced OS was mediated by reactive oxygen species (ROS) overproduction and nuclear factor erythroid 2-related factor 2 (NRF2) pathway disruption. All observed effects were contingent upon TH signaling impairment. These findings uncover novel mechanistic links between BP exposure and BFCN neurodegeneration, providing a framework for therapeutic strategies.

## 1. Introduction

Butylparaben (BP), a derivative of p-hydroxybenzoic acid, is widely employed as a preservative in different products such as cosmetics, toiletries, pharmaceuticals, and foods, among others, due to its antifungal and antimicrobial properties [1,2]. BP and other parabens are present in the environment, representing another source of human exposure [3].

BP exposure has been related to the induction of immunosuppression [4], endocrine disruption [2,5,6], reproductive toxicity [2], nephrotoxicity [7], hepatotoxicity [1], and neurotoxicity [8,9,10], among other toxic effects. Developmental BP exposure has been shown to induce cognitive disorders in adult rats [11], and repeated BP treatment has been related to memory decline in adult zebrafish [8,10]. However, the underlying processes responsible for these alterations remain unknown.

BP-induced cognitive dysfunction has been hypothesized to occur through neurotransmission disruption and neurodegeneration [10,11]. Cholinergic transmission regulates learning and memory processes [12]. Basal forebrain (BF) cholinergic neurons constitute the main cholinergic population area in the central nervous system, projecting to regions such as the hippocampus (HC) and frontal cortex (FC) to regulate cognitive function [12,13]. Maintaining basal forebrain cholinergic neuron (BFCN) viability and cholinergic neurotransmission is essential to prevent cognitive decline, whereas BFCN neurodegeneration and/or cholinergic transmission disruption can trigger FC and HC neurodegeneration and memory dysfunction [12,14]. BP has been reported to induce primary FC neurodegeneration [9], which might be mediated in vivo through BFCN loss. Therefore, it is imperative to investigate whether BP induces cholinergic neurotransmission disruption and/or BFCN neurodegeneration as a potential origin of the observed cognitive dysfunction and neurodegeneration in associated brain regions.

BP has been reported to increase acetylcholine (ACh) levels in the adult zebrafish brain [10], which is critical for cognitive functions and cellular viability [15], and to inhibit acetylcholinesterase (AChE) activity [16], the enzyme responsible for ACh metabolism [15]. A decline of ACh levels could lead to neurodegeneration and cognitive decline, but its increment above normal levels could lead to the opposite effects [17,18]. AChE exists in different variants, including the R variant, which has a protective effect on neuronal viability, and the S variant, whose upregulation triggers cell death [19,20]. Notably, silencing AChE-S can prevent cell death [21]. Therefore, BP may disrupt cholinergic transmission and/or induce BFCN neurodegeneration, leading to the reported cognitive decline.

BP has been described to induce oxidative stress (OS) following single and repeated treatment on animal studies and cell lines [2,3,4,16,22,23], ultimately triggering cell death [23,24]. BP induces OS by promoting reactive oxygen species (ROS) production [22] and weakening antioxidant defenses, mediated by downregulation of the nuclear factor (erythroid-derived 2)-like 2 (NRF2) signaling pathway [25]. OS can induce protein denaturation, leading to the loss of function, and promote the accumulation of misfolded/aggregated toxic proteins such as phosphorylated Tau (p-Tau) and amyloid-β (Aβ) peptides, resulting in neuronal death and cognition decline [26]. OS has been suggested as a potential mechanism underlying BP-induced behavioral alterations [10]. Therefore, BP could induce OS in BFCN, potentially leading to neurodegeneration and cognitive decline.

Repeated BP treatment altered the expression of heat shock transcription factor 1 (HSF-1), the master regulator of heat shock protein (HSP) expression, as well as HSP70 and HSP16.2 in *Caenorhabditis elegans* [27] or HSP90 and HSP70 in *Mauremys sinensis* [25]. A single BP treatment has also been reported to alter the expression of the chaperone GRP78 in primary murine cortical neurons [9], which regulates the unfolded protein response (UPR) signaling pathway, promoting the refolding of misfolded proteins or their degradation to support cell survival [28]. Chaperones prevent the accumulation of misfolded toxic proteins and apoptotic cell death [26]. Heat shock proteins 70 and 90, key HSPs, facilitate the clearance of misfolded/aggregated toxic proteins like p-Tau and Aβ peptides, thereby preventing neurodegeneration; conversely, their reduction promotes the increase in levels of these proteins, neuronal death, and cognition decline [26].

Repeated BP treatment of HTR8/SVneo cells or zebrafish kidney larvae inhibits or downregulates, respectively, the protein kinase B (Akt)/phosphatidylinositol 3-kinase (PI3K)/glycogen synthase kinase 3 beta (GSK3β) signaling pathway, triggering apoptosis [7,24]. GSK3β activity is reduced through Ser9 residue phosphorylation, while its dephosphorylation enhances its activity, which is associated with Tau protein hyperphosphorylation [29,30]. Therefore, HSP dysfunction and AKT/GSK3β pathway impairment may promote p-Tau and Aβ peptide accumulation, leading to neurodegeneration and cognitive dysfunction.

Repeated BP exposure has also been described to decrease thyroid hormones (THs) and reduce type 1 iodothyronine deiodinase (D1) expression in adult rats [5,31]. THs are essential for BFCN viability maintenance and participate in regulating memory and learning processes [32,33]. However, their decrease induced BFCN loss and cognitive decline [32,33]. THs regulate ACh levels through modulating AChE activity, but the decreases in their levels impair ACh metabolism [29,34,35]. THs also regulate the expression of AChE variants, the NRF2 and AKT/GSK3β pathways, HSPs, and p-Tau and Aβ levels in BFCN [29,36]. The primary TH-metabolizing enzyme in neurons is D3 [37], and its decreased expression increases T3 levels [38]. Docking studies predict that BP could bind to thyroid hormone receptors alpha (TRα), the predominant thyroid receptor in the brain [39], and TRβ [40,41], and it induces the proliferation of GH3 rat cells, which express TH receptors, suggesting it could be an agonist of these receptors [41]. Therefore, BP may trigger cholinergic transmission dysfunction, OS, and p-Tau and Aβ peptide accumulation via HSPs dysfunction and AKT/GSK3β pathway impairment, triggering BFCN death mediated by THs activity dysfunction, which could lead to cognitive decline.

Based on the above, we hypothesized that both single and repeated BP treatment may induce THs activity dysfunction that leads to cholinergic neurotransmission alteration, OS generation, and p-Tau and Aβ peptide accumulation, ultimately triggering BFCN loss. To test this hypothesis, we treated SN56 wild-type or transfected cells (used as an in vitro BFCN model) with BP (0.1 µM to 80 µM). Cells were silenced for *AChE*, *GSK3β*, microtubule-associated protein Tau (*Tau*), and/or β-amyloid precursor protein (*βApp*), with or without thyroxine (T3; 15 nM), recombinant HSP70 (rHSP70; 30 μM), and/or NAC (1 mM). This experimental strategy systematically investigates the molecular pathways driving the BP-mediated degeneration of BFCN that may contribute to cognitive decline, while also identifying potential preventative and therapeutic strategies.

## 2. Materials and Methods

### 2.1. Reagents

Butylparaben (≥99%), acetylcholine, acetylthiocholine, dibutyryl-cAMP, dimethyl sulfoxide (DMSO), dithionitrobenzoic acid (DTNB), 3-(4,5-dimethylthiazol-2-yl)-2,5-diphenyltetrazolium bromide (MTT), N-acetylcysteine (NAC), poly-L-lysine, retinoic acid, triiodothyronine and tetraisopropylpyrophosphoramide (iso-OMPA) were purchased from Sigma-Aldrich (Madrid, Spain). All other chemicals used were of reagent grade and the highest available purity.

### 2.2. Culture Conditions

The SN56 cell line, derived from cholinergic septal neurons [42], kindly gifted by Professor Laura Calzà (CIRI-SDV and Fabit, University of Bologna), served as an experimental model of BFCN to investigate the toxic effects of BP on this neuronal population and the underlying mechanisms. Cells were maintained in Dulbecco’s Modified Eagle’s Medium (DMEM; Sigma-Aldrich, Madrid, Spain) supplemented with penicillin/streptomycin, 10% fetal bovine serum (FBS), 2 mM L-glutamine, and 1 mM sodium pyruvate. Cultures were incubated at 37 °C under 5% CO_2_, and the medium was replaced every 48 h [43]. To induce morphological maturation and elevate choline acetyltransferase activity and acetylcholine levels by 3- to 4-fold, cells were differentiated by culturing for 72 h with 1 mM dibutyryl-cAMP and 1 µM retinoic acid [44,45]. This differentiation step is critical, as neurotoxic xenobiotics exhibit greater effects on cholinergic pathways in differentiated cells [44,45]. Mycoplasma contamination was routinely assessed and excluded using the LookOut^®^ Mycoplasma Detection Kit (Sigma-Aldrich, Madrid, Spain).

To investigate the neurotoxic mechanisms of BP, SN56 cells were seeded at 1 × 10^6^ cells/well in 6-well plates and subjected to comprehensive analysis. We quantified (1) cellular content of ACh, HSP70, NRF2, protein carbonyls, malondialdehyde (MDA), hydrogen peroxide (H_2_O_2_), superoxide dismutase 1 (SOD1), heme oxygenase 1 (HO1), phosphorylated GSK3β (p-GSK3β, Ser9), Aβ_1-42_, and Tau; (2) AChE activity; (3) gene expression of *AChE* splice variant, *AChE*, *βApp*, *GSK3β*, and *Tau*; and (4) consequences following RNAi-mediated knockdown of *βApp*, *Tau*, *GSK3β*, and *AChE*.

Treatments tested our central hypothesis through daily administration of BP (0.1–80 μM) for 1 or 14 days, with the following parallel combinatorial interventions: T_3_ (15 nM), NAC (1 mM), and/or rHSP70 (30 μM). BP stock aliquots were progressively diluted in sterile culture medium to generate working concentrations, ensuring final treatment solutions contained 0.1% DMSO as carrier solvent. All experimental conditions included matched vehicle controls and were replicated in ≥3 independent wells per treatment group.

Parabens are rapidly absorbed, metabolized by esterases, and excreted primarily in urine (approximately 70%). However, a fraction remains unmetabolized and may accumulate in tissues, with detection persisting up to 72 h post-exposure, particularly for BP, due to its slower metabolism [1,2,22,46]. These compounds were detected in several biological fluids (blood, amniotic fluid, breast milk, cord blood, and urine) and tissues (breast tumors and adipose tissue) [9,47,48]. BP concentrations in human urine range from 0.2 µg/L (0.001 µM) to 1240 µg/L (8 µM) [49]. Therefore, the human population is widely exposed to this and other parabens, posing a high risk to human health.

Repeated BP exposure has been shown to induce memory impairment at concentrations of approximately 0.6–6 µM in adult zebrafish [8]. In vitro studies report BP-induced cell death at 15 µM (IC50: 53 µM) in HEK293T cells [7] and at 10 µM (EC50: 63 µM) in human neuroectodermal cells [50]. Given that the maximum detected urinary BP concentration is 8 µM, coupled with its slow metabolism, potential tissue accumulation, and the lag time between exposure and urinary measurement, the actual tissue concentrations, particularly in occupationally exposed individuals (e.g., industrial handlers), may be significantly higher than those detected in urine.

Based on these findings and the potential exposure levels in humans, we selected a concentration range of 0.1 µM to 80 µM to test our hypothesis. The BP concentration selected for caspase activity and MTT assays corresponded to the minimum effective dose (MED) demonstrating significant cytotoxicity following acute exposure. This concentration threshold enabled mechanistic evaluation of BP-induced neurotoxicity, including (1) Aβ and p-Tau peptide accumulation, (2) caspase-3/7 activation, and (3) dose-dependent viability reduction. Concurrently, NAC and rHSP70 concentrations were optimized to their most efficacious therapeutic doses (MTDs) for maximal attenuation of these pathological effects.

### 2.3. TRα Activity Determination

Nuclear extracts were analyzed for TRα activation using the Thyroid Hormone Receptor Alpha Transcription Factor Activity Assay Kit (TFAB00173; Assay Genie, Dublin, Ireland) according to the manufacturer’s specifications. Total protein concentration was quantified using the BCA Protein Assay Kit (Thermo Fisher Scientific, Madrid, Spain).

The assay principle relies on the specific binding of activated TRα from treated samples to an immobilized DNA sequence containing the TRα consensus binding site. Detection occurs through a two-step immunoreaction: (1) primary antibody binding to a conformational epitope exposed only in DNA-bound, activated TRα; (2) HRP-conjugated secondary antibody-mediated color development using TMB (3,3′,5,5′-tetramethylbenzidine) substrate.

Absorbance at 450 nm was measured using a Fluoroskan FL microplate reader (Thermo Fisher Scientific). Data were normalized to nuclear protein content and expressed as percentage of untreated controls.

### 2.4. Quantification of Acetylcholine Levels

ACh release into the culture medium was quantified using a commercial colorimetric/fluorimetric assay kit (Abcam, Cambridge, UK) following established methodology [51]. Culture medium samples were collected 24 h post-treatment and centrifuged at 800× *g* to remove cellular debris. The supernatant was lyophilized and subsequently reconstituted in 50 μL of choline assay buffer before storage at −80 °C until analysis.

For ACh determination, 50 μL aliquots of processed samples were combined with AChE, choline probe, enzyme mix, and reaction solution (50 μL) containing choline assay buffer, following the manufacturer’s protocol. All samples were analyzed in triplicate, with the complete experiment replicated three times independently. Fluorescence measurements (λex = 535 nm; λem = 587 nm) were converted to ACh concentrations (pmol/well) using a choline standard curve generated for each assay run.

### 2.5. Quantification of AChE Activity

AChE activity was assessed using a spectrophotometric method based on the Ellman assay [52], with modifications introduced by Härtl et al. [53] and Zimmermann et al. [54]. Cell lysate supernatants (10 µL) were incubated in 96-well plates with Ellman’s reaction buffer containing 100 µM iso-OMPA, a specific inhibitor of butyrylcholinesterase, to ensure selective measurement of AChE activity. The enzymatic reaction was initiated by the simultaneous addition of 500 µM 5,5′-dithiobis-(2-nitrobenzoic acid) and 1 mM acetylthiocholine iodide as substrate.

Absorbance at 412 nm was monitored kinetically over 30 min at 37 °C using a microplate reader, with measurements taken at 1 min intervals. All experimental conditions were analyzed in triplicate to ensure reproducibility. AChE activity was calculated from the linear portion of the reaction curve and normalized to total protein content, expressed as nmol of hydrolyzed substrate per hour per mg of protein.

### 2.6. Assessment of Oxidative Stress

To evaluate oxidative stress parameters, we quantified protein carbonylation, H_2_O_2_ levels, and MDA formation as markers of protein oxidation, ROS generation, and lipid peroxidation, respectively. These analyses were performed using commercially available assays from Abcam: the Lipid Peroxidation MDA Assay Kit (ab233471), Hydrogen Peroxide Assay Kit (ab102500), and Protein Carbonyl Content Assay (ab126287), following the manufacturer’s standardized protocols.

For each oxidative stress marker, cell pellets containing 1 × 10^6^ cells were homogenized in ice-cold lysis buffers provided with the respective kits. Processed samples and appropriate standards were then aliquoted into 96-well plates, following assay-specific requirements. Spectrophotometric measurements were conducted using a Thermo Fisher Fluoroskan FL microplate reader, with absorbance readings performed at distinct wavelengths for each analyte: 370 nm for protein carbonyls, 572 nm for H_2_O_2_, and 532 nm for MDA quantification.

The oxidative stress biomarkers were quantified using standard curves generated for each assay, with results expressed in standardized units: H_2_O_2_ concentrations as nmol/mL. At the same time, protein carbonyl and MDA levels were normalized to total protein content and reported as nmol/mg protein. This comprehensive approach allowed for simultaneous evaluation of multiple oxidative damage pathways under consistent experimental conditions, ensuring comparability across biomarkers.

### 2.7. Quantification of Beta-Amyloid and Tau Peptide

Following treatment, culture medium from both treated and control samples was collected from 6-well plates and centrifuged at 3000× *g* for 10 min at 4 °C to remove cellular debris. The cultures were then washed with phosphate-buffered saline (PBS; Sigma-Aldrich, Madrid, Spain), and adherent cells were scraped using 100 µL of ice-cold homogenization buffer (50 mM Tris, pH 7.6, 1 mM EDTA, 150 mM NaCl, 1% Triton X-100, and supplemented with phosphatase and protease inhibitors; Sigma-Aldrich, Madrid, Spain). The lysates were incubated on ice for 5 min and subsequently clarified by centrifugation at 14,000× *g* for 10 min at 4 °C, after which, the supernatant was aliquoted and stored for further analysis.

P-Tau (KMB7041) and Aβ_1-42_ (KMB3441) levels were determined in culture medium and cell lysates, respectively, using commercially available ELISA kits (Invitrogen, Madrid, Spain) following the manufacturer’s protocol. Briefly, 100 µL of samples or standards were loaded in duplicate onto 96-well plates and incubated for 2 h at room temperature. After washing, 100 µL of detection antibody was added to each well and incubated for 1 h, followed by another wash step and a 30 min incubation with 100 µL of horseradish peroxidase (HRP)-conjugated secondary antibody. Following a final wash, stabilized chromogen was added, and the plates were incubated for 20–30 min in the dark at room temperature. The enzymatic reaction was terminated by adding 100 µL of stop solution, and absorbance was measured at 450 nm using a Thermo Fisher Fluoroskan FL microplate reader (Madrid, Spain). Raw concentrations (pg/mL) were normalized to total protein content (µg/mL) to yield final values expressed as pg/µg, ensuring comparability across samples.

### 2.8. Quantification of Target Proteins

Following PBS washing (pre-chilled), cells were mechanically detached and lysed in RIPA buffer (Thermo Scientific, Madrid, Spain) supplemented with a protease inhibitor cocktail. The lysates were centrifuged at 10,000× *g* for 10 min at 4 °C, removing cellular debris. Clarified supernatant was carefully aspirated for downstream studies. Protein concentration was determined using a BCA kit (Thermo Fisher Scientific, Madrid, Spain).

The expression levels of p-GSK3β (Ser9), HSP70, SOD1, HO1, and NRF2 were quantified using commercially available ELISA kits (MBS9501465, MBS760601, MBS451661, MBS267777, and MBS776676, respectively; MyBioSource, San Diego, CA, USA), strictly adhering to the producer’s protocols. To ensure specificity, negative controls were included for each target protein. Data normalization was determined using cellular protein content and is presented as nanograms of target protein per milligram of total protein (ng/mg).

### 2.9. Gene Expression Measurement

Total RNA was isolated and complementary DNA (cDNA) synthesized using established methodologies [55]. Gene expression profiling was performed using validated primer sets (SuperArray Bioscience, Frederick, MD, USA) targeting key transcripts: *AChE* (PPM35356A), *βApp* (PPM37085A), *Gsk3β* (PPM03380C), *Tau* (PPM24640A), and the reference gene *β-actin* (PPM02945B). Additional AChE-specific primers (*AChE-R*/*S*) were included as previously described [56]; Table 1.

Quantitative PCR amplification was carried out in a CFX96 thermocycler (Bio-Rad, Madrid, Spain) using SYBR Green master mix (PA-012; SuperArray Bioscience). The thermal profile consisted of an initial denaturation at 95 °C for 10 min, followed by 40 cycles of denaturation (95 °C, 15 s) and annealing/extension (72 °C, 30 s). All reactions were performed in technical triplicates with appropriate negative controls.

Normalization of expression data was performed against β-actin as a reference gene and analyzed using the comparative Ct method (2^−ΔΔCt^). Relative quantification of transcript levels was calculated as fold-change values relative to control conditions, following established normalization procedures [57].

### 2.10. siRNA Transfection and Gene Silencing Validation

Cells were seeded at a density of 1 × 10^6^ cells per well and transfected using the HiPerfect Transfection Reagent (Qiagen, Barcelona, Spain). siRNA duplexes were designed using the HiPerformance Design Algorithm (Novartis AG, Basilea, Switzerland) and obtained from Qiagen, targeting the following murine genes: *AChE* (GS11423), *βApp* (GS11820), *Gsk3β* (GS56637), and *Tau* (GS17762). Silencing controls were carried out using the AllStars Negative Control siRNA (Qiagen, Barcelona, Spain).

After 48 h post-transfection, silencing efficiency was assessed via RT-PCR using gene-specific primers for target genes (Qiagen, Barcelona, Spain). To determine whether gene knockdown affected cellular viability, an MTT assay was conducted. Following a 24 h incubation with siRNA, cells were washed with PBS and subsequently treated with BP or control medium for either 1 or 14 days.

### 2.11. Cell Viability Determination (Caspases 3/7 and MTT Assays)

Cell viability following BP treatment was evaluated using the MTT assay, as previously described [55]. To determine whether BP exposure triggered apoptotic pathways, caspase-3/7 activity was measured using the Caspase-Glo^®^ 3/7 luminescence assay (Promega, Madrid, Spain), in accordance with the manufacturer’s protocol.

### 2.12. Statistical Analysis

All experimental conditions were assessed in at least three independent biological experiments per condition, all of them performed in triplicate, leading to an n = 9, ensuring robust and reproducible results. Data are presented as mean values ± standard error of the mean (SEM). For comparisons between treatment groups and controls, statistical significance was determined using an unpaired two-tailed Student’s *t*-test.

To evaluate the interaction between gene manipulation and treatment effects, a two-way analysis of variance (ANOVA) was applied, whereas a one-way ANOVA was employed to assess the dose-dependent impact of BP concentrations on cellular responses. Post hoc multiple comparisons were conducted using Tukey’s test, with a significance threshold set at *p* ≤ 0.05. All statistical analyses were performed using GraphPad Prism 5.0 Software Inc. (GraphPad Software, San Diego, CA, USA).

## 3. Results

### 3.1. Quantification of D3 Levels and TRα Activity and Levels

**Figure 1:**
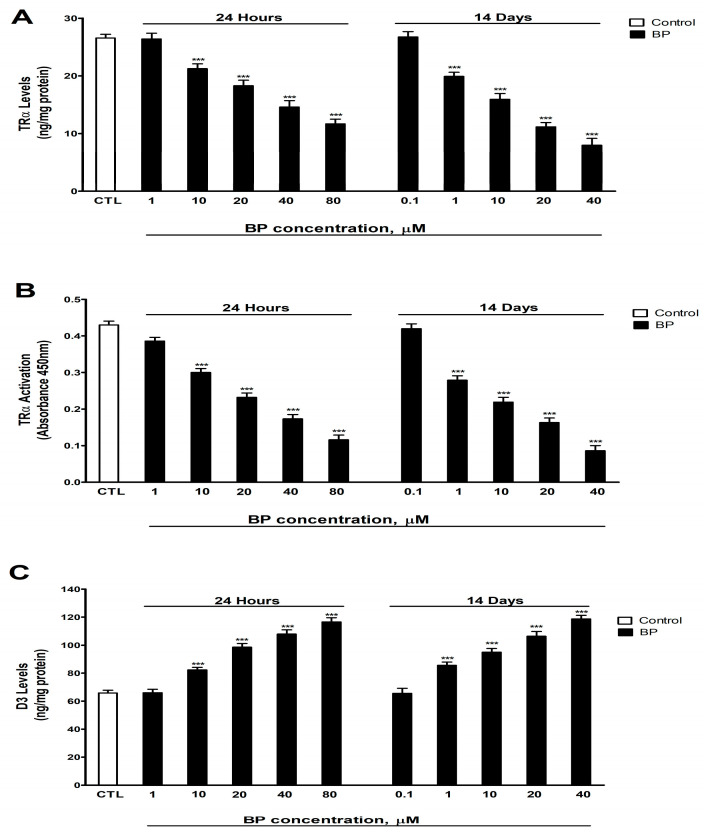
BP (0.1–80 µM) effects on (**A**) TRα levels, (**B**) TRα activation, and (**C**) D3 levels in SN56 cell homogenates after one- and fourteen-days of treatment. The mean ± SEM was obtained from data of three replicates of cultures performed three different times. *** *p* ≤ 0.001, significantly different from controls.

D3 levels and TRα activity and levels were analyzed following one- and fourteen-days of BP exposure (0.1–80 µM). BP treatment induced a significant concentration-dependent decrease in TRα levels (Figure 1A) and activity (Figure 1B) and a significant increase in D3 levels (Figure 1C). There was no statistically significant difference between controls with or without the vehicle. This corroborated that the vehicle did not affect the results.

### 3.2. Quantification of ACh Levels and AChE Activity and Variants Expression

**Figure 2:**
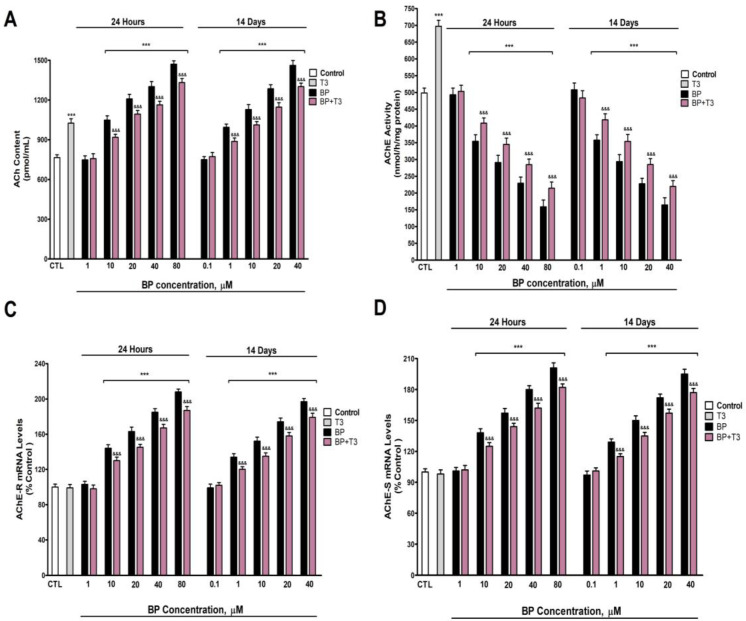
BP (0.1–80 µM) effects on (**A**) ACh content, (**B**) AChE activity, (**C**) AChE-R, and (**D**) AChE-S gene expression in SN56 cell homogenates after one- and fourteen-days of treatment. Data represents the mean ± SEM of three separate experiments from cells of different cultures, each one performed in triplicate. *** *p* ≤ 0.001 compared to control. ^&&&^ *p* ≤ 0.001 compared to BP treatment.

ACh levels in the supernatant of cell cultures were quantified following 1- and 14-days of exposure to BP at concentrations ranging from 0.1 µM to 80 µM. BP treatment induced a dose-dependent elevation in ACh levels compared to the control group, with statistically significant effects observed at concentrations ≥10 µM after 1 day and ≥1 µM after 14 days (Figure 2A). Treatment with T3 also significantly increased ACh levels. However, co-treatment with T3 and BP attenuated the BP-induced rise in ACh (Figure 2A).

Analysis of AChE enzymatic activity revealed a dose-dependent and statistically significant reduction following BP exposure (0.1–80 µM) for both 1 day (threshold: ≥10 µM) and 14 days (threshold: ≥1 µM) (Figure 2B). To exclude confounding effects from potential cytotoxicity, the AChE activity was normalized to total protein content, confirming that BP directly inhibited AChE. Treatment with T3 significantly elevated AChE activity. However, BP co-treatment with T3 partially mitigated the BP-induced suppression of AChE (Figure 2B).

The gene expression analysis of *AChE* variants revealed that BP treatment induced the dose-dependent upregulation of both *AChE-R* (Figure 2C) and *AChE-S* (Figure 2D) mRNA levels, with significant effects observed at concentrations ≥10 µM after 1 day and ≥1 µM after 14 days. In contrast, T3 treatment alone did not significantly alter the expression of either *AChE-R* (Figure 2C) or *AChE-S* (Figure 2D). Notably, co-treatment with T3 and BP partially attenuated the BP-induced overexpression of both *AChE* variants (Figure 2C,D).

### 3.3. NRF2 Pathway Assessment (HO1, SOD1, and NRF2 Protein Levels Quantification)

**Figure 3:**
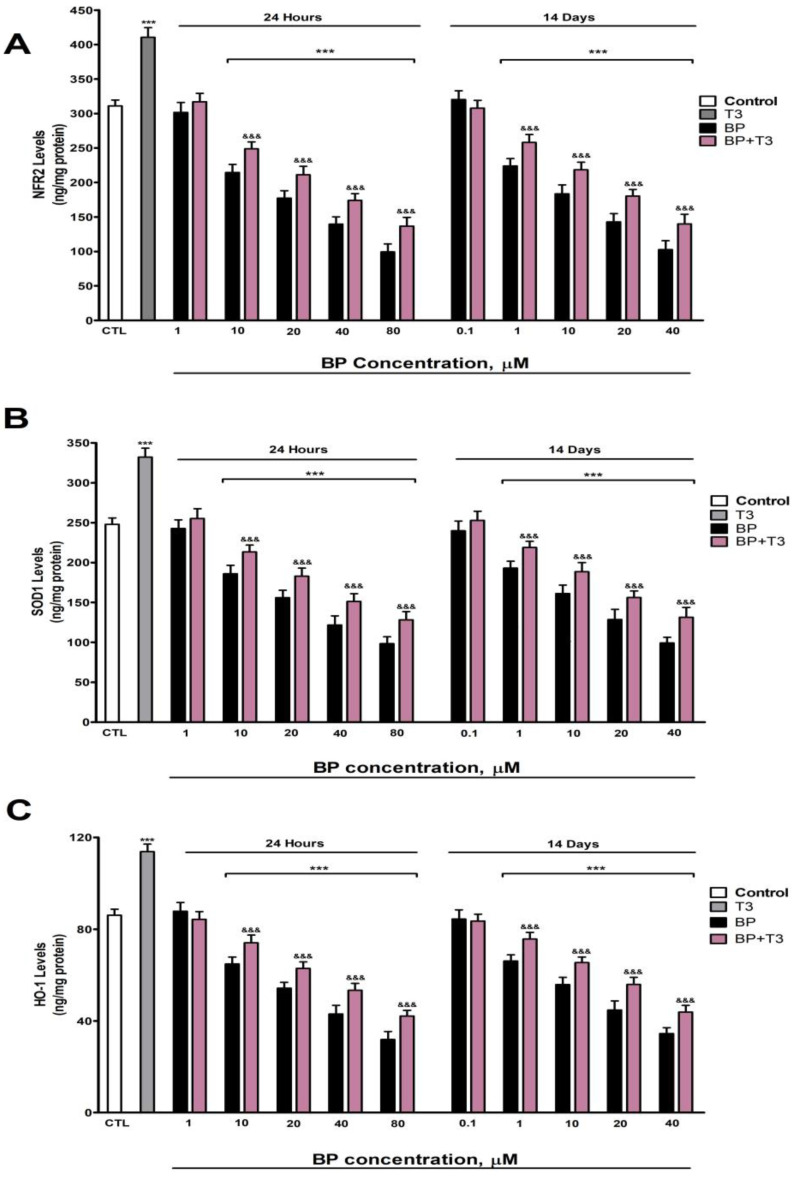
BP (0.1–80 µM) effects on (**A**) NRF2, (**B**) SOD1, and (**C**) HO1 contents in SN56 cell homogenates after one- and fourteen-days of treatment. Data represents the mean ± SEM of three separate experiments from cells of different cultures, each one performed in triplicate. *** *p* ≤ 0.001 compared to control. ^&&&^ *p* ≤ 0.001 compared to BP treatment.

NRF2, SOD-1, and HO-1 protein levels (Figure 3A, Figure 3B, and Figure 3C, respectively) showed a dose-dependent reduction following both 1-day (threshold: ≥10 µM) and 14-day (threshold: ≥1 µM) BP treatment, with progressively greater effects observed at higher concentrations. T3 treatment significantly increased the SOD-1 (Figure 3B) and HO-1 (Figure 3C) protein content in SN56 cells. T3 co-treatment with BP partially lessened the reduction in NRF2 (Figure 3A), SOD-1 (Figure 3B), and HO-1 (Figure 3C) protein content observed after BP treatment alone (Figure 3).

### 3.4. Oxidative Stress Analysis

**Figure 4:**
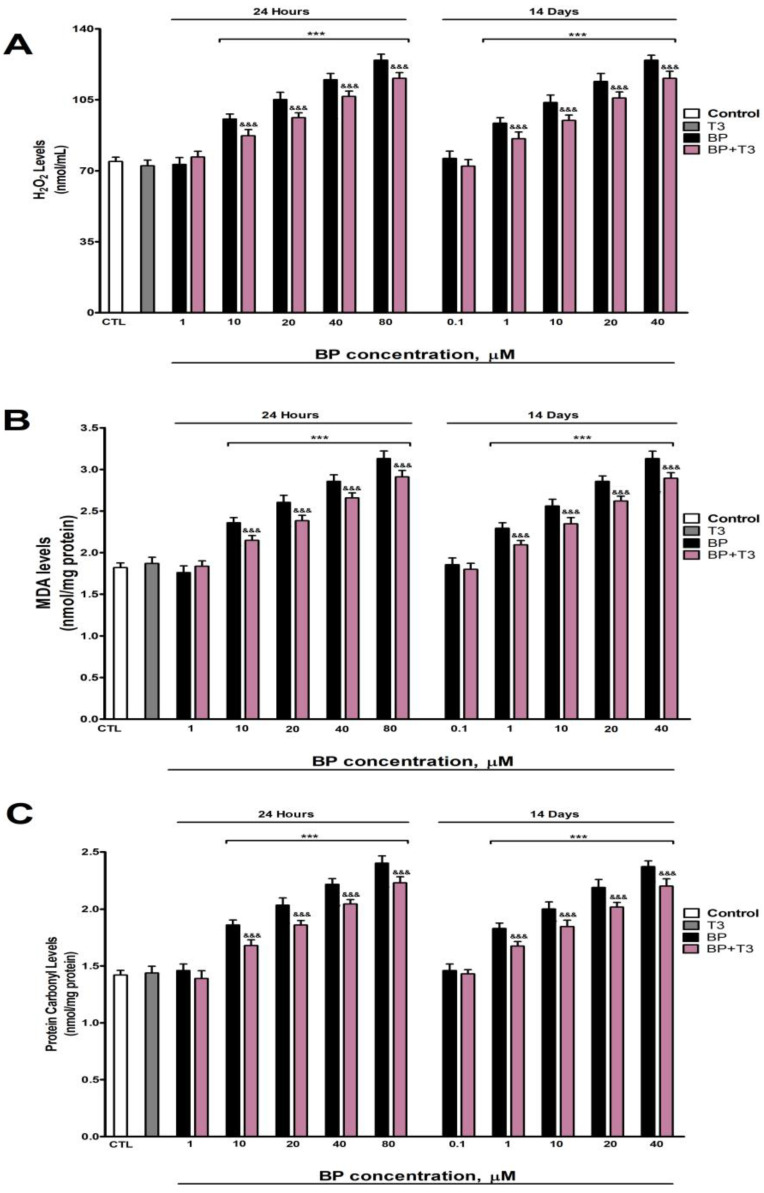
BP (0.1–80 µM) effects on (**A**) H_2_O_2_, (**B**) MDA, and (**C**) protein carbonyl contents in SN56 cell homogenates after one- and fourteen-days of treatment. Data represents the mean ± SEM of three separate experiments from cells of different cultures, each one performed in triplicate. *** *p* ≤ 0.001 compared to control. ^&&&^ *p* ≤ 0.001 compared to BP treatment.

Levels of H_2_O_2_, MDA, and protein carbonyls (Figure 4A, Figure 4B, and Figure 4C, respectively) exhibited a dose-dependent elevation following BP treatment, with significant increases observed at concentrations ≥10 µM after 1 day and ≥1 µM after 14 days. T3 treatment has not affected the H_2_O_2_ (Figure 4A), MDA (Figure 4B), and protein carbonyls (Figure 4C) content in SN56 cells. T3 co-treatment with BP partially lessened the increase of H_2_O_2_ (Figure 4A), MDA (Figure 4B), and protein carbonyl (Figure 4C) levels observed following BP exposure (Figure 4).

### 3.5. Quantification of HSP70 and p-GSK3β (Ser9) Levels

**Figure 5:**
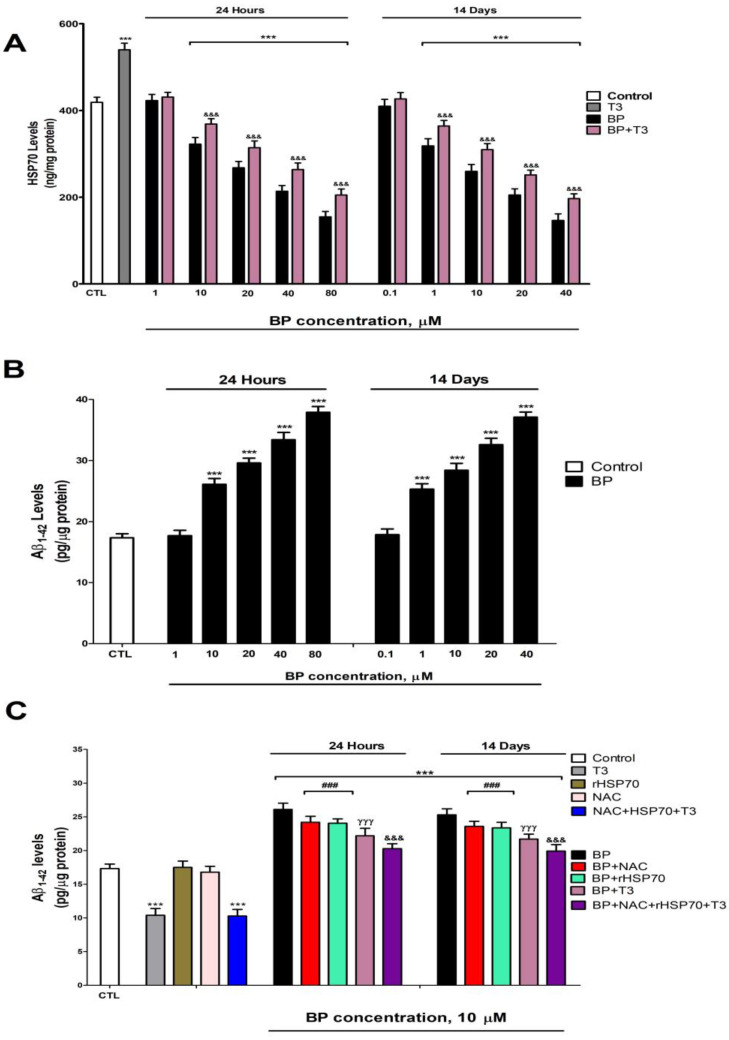
BP (0.1–80 µM) effects on (**A**) HSP70 and (**B**) Aβ_1-42_ protein content after one- and fourteen-days of treatment. Effects of treatment with BP (10 µM), NAC (1 mM), T3 (15 nM) or rHSP70 (30 µM), simultaneous NAC, T3, and rHSP70, or BP co-treatment with or without NAC, with or without T3, and with or without rHSP70 on Aβ_1-42_ protein content (**C**). Data represent the mean ± SEM of three separate experiments from cells of different cultures, each one performed in triplicate. *** *p* < 0.001 compared to control. ^###^ *p* ≤ 0.001 compared to BP treatment. ^γγγ^ *p* ≤ 0.001 compared to rHSP70 co-treated cells with BP. ^&&&^ *p* ≤ 0.001 compared to T3 co-treated cells with BP.

**Figure 6:**
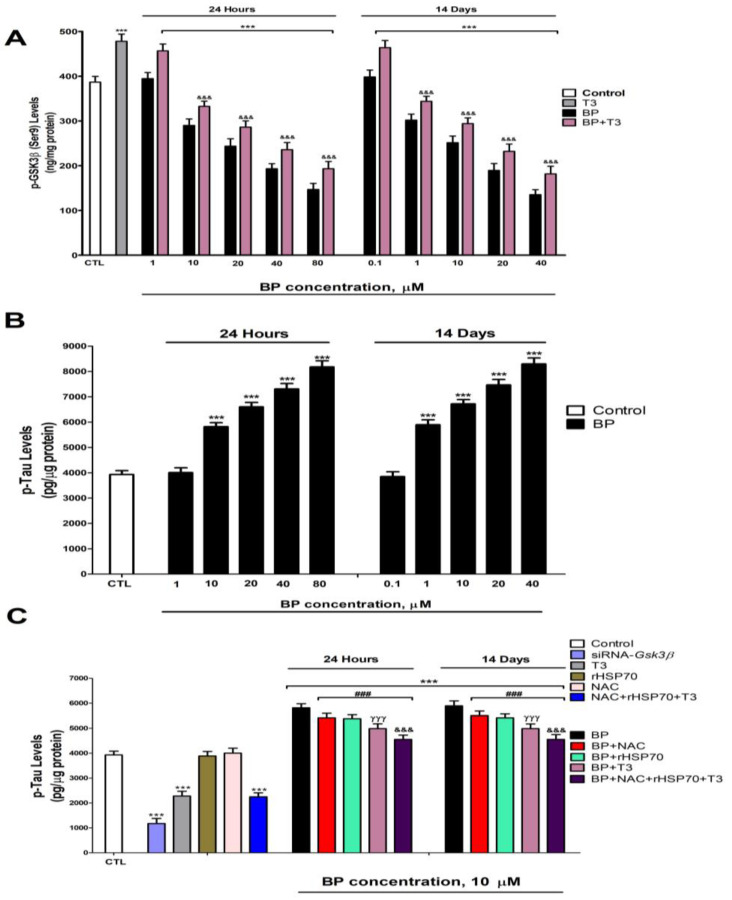
BP (0.1–80 µM) effects on (**A**) p-GSK3β (Ser9) and (**B**) p-Tau protein content after one- and fourteen-days of treatment. Effects of *Gsk3β* knockdown, treatment with BP (10 µM), NAC (1 mM), T3 (15 nM) or rHSP70 (30 µM), simultaneous NAC, T3, and rHSP70, or BP co-treatment with or without NAC, with or without T3, and with or without rHSP70 on p-Tau protein content (**C**). Data represent the mean ± SEM of three separate experiments from cells of different cultures, each one performed in triplicate. *** *p* < 0.001 compared to control. ^###^ *p* ≤ 0.001 compared to BP treatment. ^γγγ^ *p* ≤ 0.001 compared to rHSP70 co-treated cells with BP. ^&&&^ *p* ≤ 0.001 compared to T3 co-treated cells with BP.

Protein levels of HSP70 (Figure 5A) and p-GSK3β (Ser9) (Figure 6A) exhibited a dose-dependent decrease following BP treatment, with significant reductions observed at concentrations ≥10 µM after 1 day and ≥1 µM after 14 days. T3 treatment significantly increased the protein levels of HSP70 (Figure 5A) and p-GSK3β (Ser9) (Figure 6A). T3 co-treatment with BP partially lessened the protein level decrease in HSP70 (Figure 5A) and p-GSK3β (Ser9) (Figure 6A) observed after BP treatment alone (Figure 4).

### 3.6. Quantification of Tau and β-Amyloid Peptide Levels

Protein levels of p-Tau (Figure 5B) and Aβ_1-42_ (Figure 6B) showed dose-dependent increases following BP exposure, with significant effects observed at ≥1 µM after 1 day and ≥10 µM after 14 days. *Gsk3β* knockdown effectively reduced p-Tau accumulation (Figure 5C). While individual treatments with NAC, rHSP70, or T3 showed no effect on basal p-Tau (Figure 5C) or Aβ_1-42_ (Figure 6C) levels in wild-type cells, individual NAC, rHSP70, or T3 co-treatments with BP resulted in partial attenuation of the BP-induced increases. Notably, T3 showed superior protective effects compared to NAC or rHSP70 alone (Figure 5C and Figure 6C). The combined co-treatment of T3, NAC, and rHSP70 with BP produced the most pronounced reduction in both p-Tau and Aβ_1-42_ accumulation, though complete normalization was not achieved (Figure 5C and Figure 6C).

### 3.7. Gene Knockdown Validation Assessment

**Figure 7:**
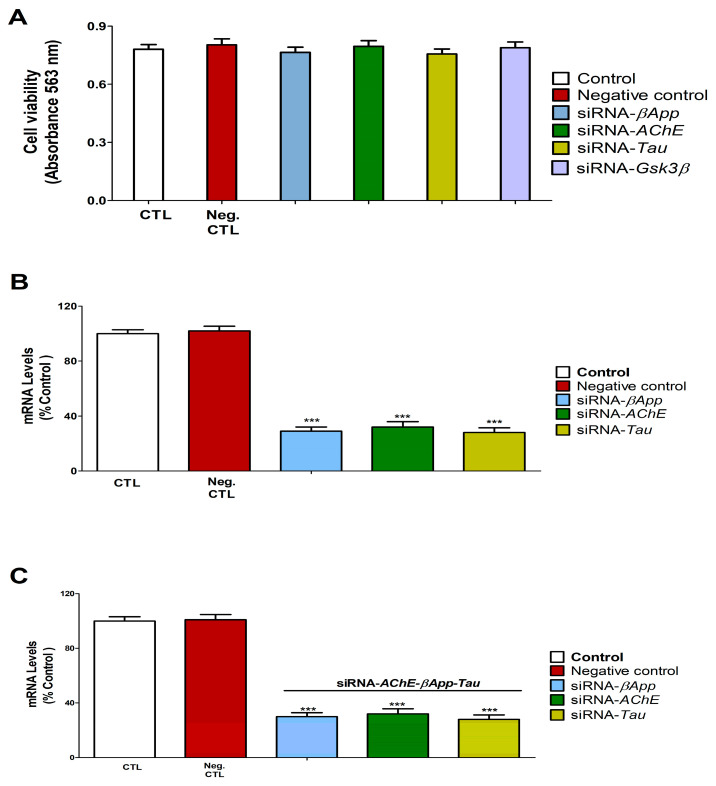
*AChE*, *Gsk3β*, *βApp*, and *Tau* silencing effect on SN56 cell viability and gene expression. Control: SN56 cells transfected without siRNA. Negative (Neg.) control: SN56 cells transfected with scrambled siRNA. *AChE*-siRNA: transfected with siRNA against *AChE*. *Gsk3β*-siRNA: transfected with siRNA against *Gsk3β*. *βApp*-siRNA: transfected with siRNA against *βApp*. *Tau*-siRNA: transfected with siRNA against *Tau*. MTT analysis shows that *AChE*, *Gsk3β*, *βApp*, and *Tau* knockout did not significantly induce cell damage after 48 h (**A**). *AChE*, *Gsk3β*, *βApp*, and *Tau* downregulation could be detected by RT-PCR analysis 48 h after transfection (**B**,**C**). Values are given as mean ± SEM of three separate experiments from cells of different cultures, each one performed in triplicate. *** *p* ≤ 0.001 compared to control.

**Figure 8:**
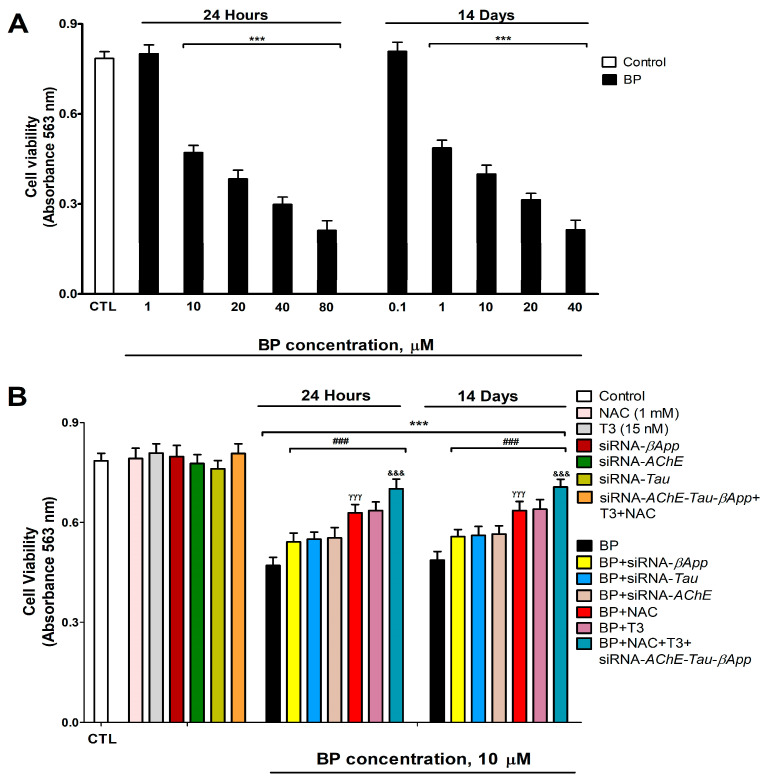
BP (0.1–80 µM) effects on SN56 cells viability (**A**). BP (10 µM) effect on wild-type or on single/simultaneous *βApp*, *Tau*, and *AChE*-silenced cells co-treated with or without T3 (15 nM) and/or NAC (1 mM) (**B**). Cell viability was determined by MTT test. Data represents the mean ± SEM of three separate experiments from cells of different cultures, each one performed in triplicate. *** *p* < 0.001 compared to control. ^###^ *p* ≤ 0.001 compared to BP treatment. ^γγγ^ *p* ≤ 0.001 compared to *AChE*-silenced cells treated with BP. ^&&&^ *p* ≤ 0.001 compared to T3 co-treated cells with BP.

Transfection of SN56 cells with individual siRNAs targeting *AChE*, *βApp*, *Gsk3β*, or Tau or combined siRNAs for *AChE*/*βApp*/*Tau* showed no effect on cell viability compared to control siRNA (Figure 7A and Figure 8B). Control siRNA transfection did not alter the expression of *AChE*, *Gsk3β*, *βApp*, or *Tau* genes (Figure 7B,C). In contrast, targeted knockdown with single siRNAs significantly reduced the expression of their respective target genes, as did the combined *AChE*/*βApp*/*Tau* siRNA treatment (Figure 7B,C).

### 3.8. Cell Viability Assessment and Caspases 3/7 Activation Determination

BP treatment induced a dose-dependent reduction in cell viability, with significant effects observed at concentrations ≥10 µM after 1 day and ≥1 µM after 14 days (Figure 8A). Neither NAC nor T3 co-treatment in wild-type cells nor the individual knockdown of *AChE*, *βApp*, or *Tau* (or combined knockdown with T3/NAC co-treatment) affected the basal cell viability (Figure 8B). However, both T3 and NAC co-treatment with BP in wild-type cells, as well as BP treatment in single-knockdown cells (*AChE*, *βApp*, or *Tau*), partially attenuated the BP-induced viability reduction. Notably, T3 or NAC co-treatment with BP provided greater protection than individual gene knockdowns (Figure 8B). The combined T3+NAC+BP treatment in triple-knockdown cells (*AChE*/*βApp*/*Tau*) showed the most pronounced attenuation of BP-induced cytotoxicity, though complete recovery was not achieved (Figure 8B). No significant differences were observed between vehicle-treated and untreated controls.

**Figure 9:**
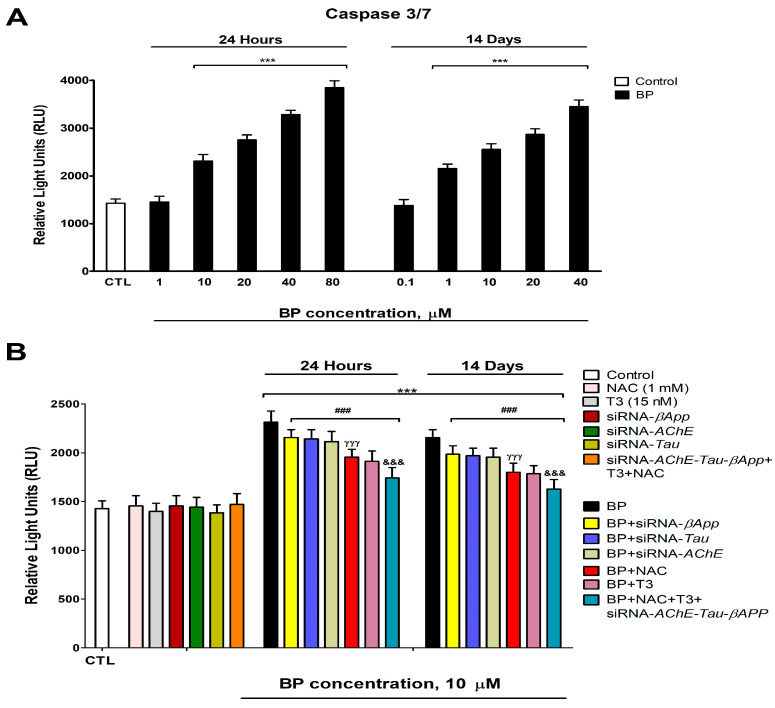
Analysis of caspases 3/7 activity after BP (0.1–80 µM) treatment in SN56 cells (**A**). Analysis of caspases 3/7 activity in BP (10 µM) wild-type or *βApp*, *Tau*, and *AChE*-silenced cells co-treated with or without T3 (15 nM) and/or NAC (1 mM) (**B**). Values are expressed as mean ± SEM of three separate experiments from cells of different cultures, each one performed in triplicate. *** *p* < 0.001 compared to control. ^###^ *p* ≤ 0.001 compared to BP treatment. ^γγγ^ *p* ≤ 0.001 compared to *AChE*-silenced cells treated with BP. ^&&&^ *p* ≤ 0.001 compared to T3 co-treated cells with BP.

Caspase 3/7 activity showed a dose-dependent increase following BP exposure, with significant activation observed at concentrations ≥10 µM after 1 day and ≥1 µM after 14 days (Figure 9A). Neither T3 nor NAC treatment in wild-type cells, the individual knockdown of *AChE*, *βApp*, or *Tau*, nor the combined T3+NAC treatment in triple-knockdown cells (*AChE*/*βApp*/*Tau*) induced caspase 3/7 activation (Figure 9B). However, both T3 and NAC co-treatment with BP in wild-type cells, as well as BP treatment in single-knockdown cells, partially attenuated BP-induced caspase activation. Notably, T3 or NAC co-treatment provided superior protection compared to individual gene knockdowns (Figure 9B). The combined T3+NAC+BP treatment in triple-knockdown cells produced the most substantial reduction in caspase activation, though complete recovery was not achieved (Figure 9B). These caspase activity findings corroborate the viability results, supporting BP-induced apoptotic cell death.

## 4. Discussion

Unique (24 h) and repeated (14 days) treatment with BP of SN56 cells decreases, in a concentration-dependent manner (starting at 10 µM or 1 µM, respectively), the levels and activity of TRα and increases D3 levels. These findings indicate that BP disrupts THs signaling in SN56 cells by impairing both the TH metabolism and downstream pathway activation. THs regulate cognitive function [32,33], so this disruption may underlie BP-induced memory and learning deficits. To our knowledge, this is the first time that these results have been reported.

Docking studies predicted that BP could bind to TRα, the principal thyroid receptors present in the brain [39], and induce the proliferation of GH3 rat cells, which express TH receptors, suggesting it could be an agonist of these receptors [41], supporting the ability of BP to bind TRα and modulate their activity. Moreover, repeated BP exposure was shown to decrease the expression of D1 in adult rats [5,31], supporting the ability of BP to reduce the metabolism of THs. The TRα activity decrease observed could be mediated through the increased metabolism of T3 due to an increase in D3 levels, a TRα downregulation, and probably through a direct blocking of THs binding to these receptors.

Unique (24 h) and repeated (14 days) treatment with BP increases, in a concentration-dependent manner (starting at 10 µM or 1 µM, respectively), the levels of ACh and the expression of *AChE* variants (R/S) and decreases AChE activity. These results show that BP produced cholinergic neurotransmission disruption. The expression disruption of the variants (R/S) of *AChE* following BP treatment has not been described until now. BP was shown to elevate ACh levels in the adult zebrafish brain [10] and inhibit AChE activity [16], supporting the data obtained. While cholinergic transmission disruption is likely mediated by AChE inhibition, we cannot exclude contributions from altered transporters that mediate its release and uptake or the enzyme choline acetyltransferase, which produces ACh synthesis [58]. ACh maintains cognitive functions, and its reduction leads to cognitive decline [15]. However, ACh levels increasing above normal levels have also been reported to alter cognitive function [17,18]. Therefore, these results may assist in explaining the cognition alterations described after BP exposure.

BP co-treatment with T3 lessens, in part, the effect observed on ACh levels, *AChE-S*/*R* variants expression, and AChE activity inhibition produced following the BP treatment alone, showing that BP alters cholinergic neurotransmission and *AChE* variants expression through TH disruption and pointing out that other mechanisms are probably also involved. THs regulate cholinergic transmission, modulating ACh levels, AChE activity, and *AChE* variants expression [33,34,35]. T3 deficiency alters ACh content, decreases AChE activity, and upregulates the AChE-R/S variants in BFCN [33]. T3 treatment was shown to increase AChE expression and activity in vitro and in vivo [33,35,59], supporting our findings. BP was shown to trigger resistance to insulin [60], and this hormone regulates ACh levels, AChE activity, and *AChE-R*/*S* variants expression [61,62,63,64,65]. Therefore, insulin signaling disruption could also mediate these alterations.

Single (24 h) and repeated (14 days) BP treatment increases, in a concentration-dependent manner (starting at 10 µM or 1 µM, respectively), protein carbonylation and lipid peroxidation, probably due to the rise in ROS levels and the decrease in the antioxidant NRF2 pathway (reduction in NRF2, SOD1, and HO1 protein content). BP was described to induce OS after single and repeated treatment in cell lines and animal studies [2,3,4,16,22,23] through ROS production [22] and the NRF2 signaling pathway downregulation [25], supporting our findings. Concomitant treatment with BP and NAC or T3 fully or incompletely attenuated BP-induced ROS elevation, protein carbonylation and lipid peroxidation, and the downregulation of the antioxidant NRF2 pathway, showing that TH disruption mediates the induction of OS produced following BP treatment. Previous studies reported that the decrease in T3 levels leads to OS due to an elevation of ROS production and the decrease in the NRF2 pathway in different cerebral regions [36,65,66,67], supporting our results. Additional mechanisms seem to be involved; in this sense, insulin resistance was described to produce OS and downregulate the NRF2 pathway [68,69]. OS induced by BP could denature the enzymes, decreasing their activity, but not presenting any effect on their protein content. BP has been described to reduce AChE activity and induce behavioral dysfunction through ROS generation [16]. Thus, the THs disruption observed after BP treatment could trigger OS, leading to cholinergic neurotransmission alteration and cognitive decline.

Single (24 h) and repeated (14 days) BP exposure increased, in a concentration-dependent manner (starting at 10 µM or 1 µM, respectively), the Aβ_1-42_ and p-Tau levels but decreased p-GSK3β (Ser9) and HSP70 protein content. To our knowledge, this is the first report of BP effects on p-Tau and Aβ_1-42_ protein content. BP repeated treatment increases the expression of HSP70 in *Caenorhabditis elegans* and *Mauremys sinensis* [25,27], supporting the idea that BP could alter HSP70 content. This opposite effect to the one observed in our study could be due to differences between species, in vitro versus in vivo model, time of exposure, doses/concentrations used, or tissue evaluated, and it is necessary to explore the reasons behind these differences. The repeated BP treatment of HTR8/SVneo cells or zebrafish kidney larvae downregulates the p-GSK3β (Ser9) [7,24], which supports our findings. The reduction in p-GSK3β (Ser9) was reported to induce NRF2 downregulation [70]. Thus, these alterations may also play a role in the NRF2 pathway downregulation. T3 co-treatment with BP partially reversed the effect observed on these targets after treatment with BP alone. The TH levels decrease was reported to enhance Aβ_1-42_ and p-Tau protein content in rat BFCN, and T3 supplementation reverses these alterations [36], supporting our results. In addition, T3 was shown to regulate Hsp70 expression, and its deficiency downregulates its expression [71] and its supplementation increases it [72], supporting the results shown. T3 deficiency decreases p-GSK3β (Ser9) in rat BFCN, and its supplementation lessens this reduction [36], supporting the data presented. However, this reversion was not complete, suggesting additional mechanisms could be involved. NRF2 pathway downregulation has been described to reduce the *Hsp* gene expression [55,73]. ROS was also reported to increase GSK3β activity [70,74]. *AChE-S* variant upregulation has been shown to produce the accumulation of Aβ_1-42_ and p-Tau [75,76]. Insulin resistance has been described to elevate the levels of Aβ_1-42_ and p-Tau and decrease p-GSK3β (Ser9) protein content [64,77]. Thus, these alterations may also play a role in mediating these effects.

Combined BP and NAC or rHSP70 treatment partially lessens the p-Tau and Aβ_1-42_ level increase. GSK3β silencing reduces p-Tau protein content. Therefore, BP increases the Aβ_1-42_ and p-Tau levels through OS generation, reduced clearance mechanisms (via HSP70 downregulation), and enhanced formation (via p-GSK3β activation), resulting from TH signaling disruption. OS has been shown to produce the denaturalization of proteins and loss of function, the accumulation of misfolded/aggregated toxic proteins such as phosphorylated-Tau (p-Tau) and amyloid-β (Aβ) peptides, neuronal death, and cognition alteration [26]. Reduced HSP70 levels promote Aβ_1-42_ and p-Tau proteins accumulation, whereas increased HSP70 expression counteracts these effects [26,78]. The GSK3β activity elevation increases p-Tau levels [30,79,80], which backs up the effects shown.

Last of all, single (24 h) and repeated (14 days) BP treatment increased, in a concentration-dependent manner (starting at 10 µM or 1 µM, respectively), triggered cell death, possibly produced by apoptosis induction since caspases were activated. BP single and repeated treatment induced apoptosis in rat spermatogenic cells and turtle liver, respectively [24,81]. Additionally, BP single treatment induced apoptotic cell death in thyroid epithelial cells starting at 66 µM [31], in HEK293T cells at 15 μM [7], in primary cortical neurons at 500 μM [9], and in HepG2 cells at 125μM [23]. However, it produced apoptosis following repeated exposure in HTR8/SVneo cells starting at a concentration of 50 µM [24] and in human neuroectodermal cells beginning at approximately 10 μM [50]. All these published data support the results presented. The differences between the concentrations at which BP starts to induce cell death may be produced through differences in the exposure time, species, and tissue from which these cell lines came or the procedure performed.

Combined BP treatment with the T3, NAC, or rHSP70 of wild-type cells or treatment with BP alone of *Tau*, *βApp*, or *AChE*-silenced cells lessens the neuronal viability reduction and neuronal death increment than that induced following unique BP exposure, showing that these mechanisms play a role in the viability reduction produced. THs maintain the BFCN viability [32,33], but their deficiency induces BFCN loss and cognitive decline [32,33]. Upregulation of the *AChE-S* variant triggers cell death [19,20], but its silencing avoids cell death [21], which suggests that this upregulation of the *AChE* variant may induce the cell viability reduction observed. Repeated BP treatment was reported to induce apoptosis in human trophoblast cells through OS generation [24]. OS generation, Aβ and Tau proteins accumulation, and HSP70 levels decrease were shown to produce BFCN death [29,30,36,80,82,83,84,85]. Therefore, all these previous reports support our findings.

The simultaneous *AChE*, *Tau*, and *βApp* knockdown of cells co-treated with T3, NAC, and rHSP70 triggers the highest reversion in cell death induction compared to that produced in wild-type cells following BP single exposure. However, it failed to completely prevent the induction of cell death, pointing out that additional mechanisms may contribute to this effect. Insulin resistance, which BP produces, was reported to produce BFCN death [65]. BP was reported to induce apoptosis through GSK3β activation [7,24], which also regulates the Wnt signaling pathway [86]. Wnt pathway downregulation has been related to BFCN loss and cognitive dysfunction [87]. BP developmental exposure was shown to reduce the brain-derived neurotrophic factor (BDNF) in rat brain [11]. BDNF plays a critical neuroprotective role in BFCN survival, with its deficiency leading to apoptotic cell death [88]. Thus, the aforementioned mechanisms may additionally contribute to the observed neuronal cytotoxicity.

## 5. Conclusions

To summarize all the information presented, single (24 h) and repeated (14 days) BP treatment induced (starting at 10 µM or 1 µM, respectively) THs signaling disruption, triggering cholinergic neurotransmission dysfunction through AChE inhibition and SN56 cell death. BP triggered cell death through p-Tau and Aβ proteins accumulation, OS generation mediated by NRF2 pathway activity reduction and ROS accumulation, AChE-S upregulation, and HSP70 levels reduction. More studies are necessary to determine the mechanisms through which BP produces BFCN death and to corroborate whether they are produced in vivo and mediate the cognitive decline produced by this compound. This research is relevant since it provides additional information on BP neurotoxicity, specifically on cholinergic neurotransmission disruption and BFCN neurodegeneration mechanisms, which are probably involved in the cognitive decline produced, and new tools that could be helpful to treat these effects.

## Figures and Tables

**Table 1:** Primers used for quantitative real-time PCR analyses.

Abbreviation	Gene	Forward (F) and Reverse (R) Primers
AChE-S	Acetylcholinesterase	F-ctgaacctgaagcccttagag R-ccgcctcgtccagagtat
AChE-R	Acetylcholinesterase	F-gagcagggaatgcacaag R-ggggaggtaaagaagagag

## Data Availability

The original contributions presented in this study are included in the article. Further inquiries can be directed to the corresponding authors.

## Abbreviations

The following abbreviations are used in this manuscript:

Aβ	Amyloid-β
Aβ_1-42_	Amyloid-β Peptide 1-42
ACh	Acetylcholine
AChE	Acetylcholinesterase
AChE-R	Acetylcholinesterase “Readthrough” Variant
AChE-S	Acetylcholinesterase Synaptic Variant
AKT	Protein Kinase B (PKB)
ANOVA	Analysis of Variance
βApp	β-Amyloid Precursor Protein
BDNF	Brain-Derived Neurotrophic Factor
BF	Basal Forebrain
BFCN	Basal Forebrain Cholinergic Neurons
BP	Butylparaben
cAMP	Cyclic Adenosine Monophosphate
cDNA	Complementary DNA
D1	Type 1 Iodothyronine Deiodinase
D3	Type 3 Iodothyronine Deiodinase
DMSO	Dimethyl Sulfoxide
DTNB	Dithionitrobenzoic Acid
FC	Frontal Cortex
FBS	Fetal Bovine Serum
GRP78	Glucose-Regulated Protein 78
GSK3β	Glycogen Synthase Kinase 3 beta
HC	Hippocampus
HO1	Heme Oxygenase 1
HRP	Horseradish Peroxidase
HSF-1	Heat Shock Transcription Factor 1
HSP	Heat Shock Protein
HSP70	Heat Shock Protein 70
MDA	Malondialdehyde
MED	Minimum Effective Dose
MTD	Maximum Tolerated Dose
MTT	3-(4,5-Dimethylthiazol-2-yl)-2,5-Diphenyltetrazolium Bromide
NAC	N-Acetylcysteine
NRF2	Nuclear Factor (erythroid-derived 2)-like 2
OS	Oxidative Stress
PBS	Phosphate-Buffered Saline
p-GSK3β (Ser9)	Phosphorylated GSK3β (Serine 9)
PI3K	Phosphatidylinositol 3-Kinase
p-Tau	Phosphorylated-Tau
rHSP70	Recombinant Heat Shock Protein 70
RNAi	RNA Interference
ROS	Reactive Oxygen Species
siRNA	Small Interfering RNA
SOD1	Superoxide Dismutase 1
T3	Triiodothyronine
Tau	Microtubule-Associated Protein Tau
TMB	3,3′,5,5′-Tetramethylbenzidine
TRα	Thyroid Hormone Receptor Alpha
TRβ	Thyroid Hormone Receptor Beta
THs	Thyroid Hormones
UPR	Unfolded Protein Response
